# Sadness is unique: neural processing of emotions in speech prosody in musicians and non-musicians

**DOI:** 10.3389/fnhum.2014.01049

**Published:** 2015-01-30

**Authors:** Mona Park, Evgeny Gutyrchik, Lorenz Welker, Petra Carl, Ernst Pöppel, Yuliya Zaytseva, Thomas Meindl, Janusch Blautzik, Maximilian Reiser, Yan Bao

**Affiliations:** ^1^Institute of Medical Psychology, Ludwig-Maximilians-UniversitätMunich, Germany; ^2^Human Science Center, Ludwig-Maximilians-UniversitätMunich, Germany; ^3^Parmenides Center for Art and SciencePullach, Germany; ^4^Institute of Musicology, Ludwig-Maximilians-UniversitätMunich, Germany; ^5^Department of Psychology and Key Laboratory of Machine Perception (MoE), Peking UniversityBeijing, China; ^6^Institute of Psychology, Chinese Academy of SciencesBeijing, China; ^7^Moscow Research Institute of PsychiatryMoscow, Russia; ^8^Prague Psychiatric Centre, 3rd Faculty of Medicine, Charles University in PraguePrague, Czech Republic; ^9^Institute of Clinical Radiology, Ludwig-Maximilians-UniversitätMunich, Germany

**Keywords:** functional magnetic resonance imaging, language processing, prosody, basic emotions, musical training, temporal processing

## Abstract

Musical training has been shown to have positive effects on several aspects of speech processing, however, the effects of musical training on the neural processing of speech prosody conveying distinct emotions are yet to be better understood. We used functional magnetic resonance imaging (fMRI) to investigate whether the neural responses to speech prosody conveying happiness, sadness, and fear differ between musicians and non-musicians. Differences in processing of emotional speech prosody between the two groups were only observed when sadness was expressed. Musicians showed increased activation in the middle frontal gyrus, the anterior medial prefrontal cortex, the posterior cingulate cortex and the retrosplenial cortex. Our results suggest an increased sensitivity of emotional processing in musicians with respect to sadness expressed in speech, possibly reflecting empathic processes.

## Introduction

Musical training is associated with changes in cognitive and affective processing (Barrett et al., 2013). Musicians exhibit different expressions of musical emotion (Juslin and Laukka, 2003), and show stronger emotional experience in response to music (Blood and Zatorre, 2001). Musicians possess higher skills for the recognition of emotions expressed in music (e.g., Bhatara et al., 2011), and they differ from non-musicians in the processing of the sadness and fear conveyed in music (Park et al., 2014). However, the effects of musical training are not limited to the musical domain, and in particular certain aspects of speech processing have been shown to benefit from musical training (Thompson et al., 2004; Hyde et al., 2009; Lima and Castro, 2011; Patel, 2011, 2014). Musicians show improved performance in the encoding of speech sounds (Musacchia et al., 2007; Wong et al., 2007; Strait et al., 2009a,b), in detecting speech in noise (Strait and Kraus, 2011a), in extracting rhythmical patterns in auditory sequences (Su and Pöppel, 2012), and in processing pitch in speech (Moreno and Besson, 2005; Magne et al., 2006; Besson et al., 2007; Musacchia et al., 2007; Chandrasekaran and Kraus, 2010). Moreover, musicians seem to possess advantages in processing speech prosody (Thompson et al., 2004; Lima and Castro, 2011) and extra-linguistic properties such as the emotional content of speech (Nilsonne and Sundberg, 1985; Schön et al., 2004; Chartrand and Belin, 2006; Magne et al., 2006).

The advantages musicians exhibit in both music and speech processing have been explained by enhanced acoustic skills that musicians acquire through continuous training (Patel, 2003; Chartrand et al., 2008). The transfer effect from musical training to speech processing is assumed to be due to acoustic and rhythmic similarities between the two functional domains (Besson et al., 2011; Strait and Kraus, 2011b; Jäncke, 2012). Specifically in the communication of affect, music and speech share strong similarities, which has motivated the proposition of a shared “emotional protolanguage” of music and speech (Thompson et al., 2012). In order to express emotions, both music and speech make use of the same or similar acoustic elements such as timbre or pitch (Patel, 2003; Besson et al., 2007; Chartrand et al., 2008). Similarities between music and speech are also observed in the temporal domain as musical and verbal expressions use “temporal windows” of a few seconds within which musical motives or speech utterances are represented (Pöppel, 1989, 2009).

These strong associations between music and speech have also been observed on the neural level. Similarities have been found in brain networks active during processing of both music and language (Maess et al., 2001; Levitin and Menon, 2003; Brown et al., 2004; Koelsch et al., 2004; Abrams et al., 2011; Zatorre and Schönwiesner, 2011; Escoffier et al., 2013; Frühholz et al., 2014), and it has been assumed that the communication of emotion in both domains may be based on the same neural systems associated with social cognition, including the medial superior frontal gyrus (SFG) and the anterior cingulate cortex (ACC; Escoffier et al., 2013). Similar to music, processing of emotional speech prosody has traditionally been associated with right hemispheric activation (Schirmer and Kotz, 2006; Wildgruber et al., 2006) but this view has recently been challenged by multi-phase models that assume several stages to be involved in emotional prosody processing recruiting both the left and the right hemisphere (e.g., Brück et al., 2011; Kotz and Paulmann, 2011; Witteman et al., 2012; Grandjean and Frühholz, 2013; Kotz et al., 2013). The network of brain areas involved in processing emotional prosody is assumed to mainly consist of the primary auditory cortices, the superior temporal gyrus (STG) and the inferior frontal gyrus, as well as subcortical regions including the amygdala and the hippocampus (Ethofer et al., 2012; Frühholz et al., 2012, 2014; Frühholz and Grandjean, 2013a; Kotz et al., 2013; Belyk and Brown, 2014).

Music training has been shown to alter the neural processing of music presumably based on functional and structural changes in the musician’s brain (Hyde et al., 2009; Kraus and Chandrasekaran, 2010). Are the transfer effects of musical training on speech prosody processing also observable on the neural level? Research has been supportive of this view and it has been suggested (Besson et al., 2011; Strait and Kraus, 2011b; Patel, 2014) that intense and continuing musical training leads to structural and functional changes of the brain that advance cognitive processes and increases sensitivity to acoustic features in music processing (Besson et al., 2011; Strait and Kraus, 2011b) which may subsequently also improve speech and specifically prosody processing. A number of studies have described differences between musicians and non-musicians in speech and prosody processing on the neural level (see Wong et al., 2007; Strait et al., 2009a,b; Patel, 2014). However, these studies have investigated the advantages in musicians compared to non-musicians on the level of subcortical auditory processing (Kraus and Chandrasekaran, 2010). To our knowledge, no brain imaging study has to date explicitly investigated the effects of musical training on cortical activation patterns in response to emotions conveyed in speech prosody. In line with previous studies showing that individual differences, such as stable personality traits, and also acquired musical expertise (Park et al., 2013, 2014), alter the neural responses to musically conveyed emotions such as sadness and fear, we aimed at identifying a potential cross-modal effect of musical training on the neural processing of speech prosody conveying different emotions. We expected musical training to be associated with an enhanced competence of emotional recognition, and distinctive differences in neural responses to emotional speech prosody.

## Methods

### Participants

Twenty four healthy volunteers participated in the study. Twelve were non-musicians (7 female, mean age = 19.00, SD = 0.60) who had no previous musical training and did not play any instruments, and 12 were musicians (7 female, mean age = 20.25, SD = 1.76 years) who had received formal music training (mean years of training = 13.83, SD = 2.58 years) in a variety of musical instruments (stringed instruments: 29%, accordion: 24%, piano: 35%, flute 12%). All participants were right-handed. All of them were German native speakers. None of them had a record of neurological or psychiatric illness, head trauma or psychoactive substance abuse, or had contraindications for MRI (e.g., pacemaker implant, pregnancy). Musicians and non-musicians did not differ in general health (GHQ-12, German Version by Linden et al., 1996), (independent *t*-test: *t*_(21)_ = 1.88, *p* > 0.05) or general intelligence (*t*_(22)_ = −0.65, *p* > 0.05). There was no difference between the groups in mood, measured by the “Delighted-Terrible Scale” (Andrews and Withey, 1976), before (Mann-Whitney U-test: *z* = 1.17, *p* > 0.05), or after the experiment (*z* = −0.06, *p* > 0.05), also there was no differences within neither the non-musician (Mann-Whitney U-test: *z* = 0.46, *p* > 0.05) nor the musician (*z* = 1.38, *p* > 0.05) group before and after the experiment. The study was performed in accordance to the Code of Ethics of the World Medical Association (Declaration of Helsinki) and was approved by the ethics committee of the Medical Faculty of the University of Munich. All participants signed an informed consent.

### Material

Items from the Berlin Database of Emotional Speech (Burkhardt et al., 2005) were used for the study. The database includes pre-evaluated semantically neutral sentences spoken in German in six different emotional tones (happiness, sadness, fear, disgust, boredom, neutral) by five different male and female actors. For the present study, sentences spoken by both male and female voices with three different emotional intonations conveying happiness, sadness and fear were selected. Neutral sentences spoken with a neutral intonation served as the control condition. The stimuli set has been evaluated for correct identification rates and naturalness of expression (Burkhardt et al., 2005) and for the present study, only stimuli with high values for correct detection (>65%) and naturalness (>65%) were chosen. To provide comparable and relatively long duration times, several original recordings of a given emotional quality by the same speaker were combined to last about 21 s each.

### Experimental procedure

During scanning, participants listened to the stimuli binaurally via pneumatic, noise attenuating and non-magnetic headphones. Sound level was individually adjusted to be comfortable, and light was dimmed to suppress further visual stimulation. The participants listened passively to the sentences and were asked to keep their eyes closed during the experiment.

During three measurement sessions (runs) three emotional qualities (happiness, sadness, fear) and a control condition (neutral) were presented twice (same sentences and same emotional intonation but spoken by a female and a male speaker respectively). In total, six iterations (trials) of each emotion were presented. The different conditions were presented under computer control in a pseudo-randomized order. To control for order effects, two versions of stimuli sequences were created and participants were randomly assigned to either one of them. Each stimulation-interval was followed by a pause. After scanning, participants listened to the set of stimuli again and were asked to identify the expressed emotion after each sentence by selecting an emotion from a provided list (happiness, fear, anger, disgust, sadness, surprise, neutral) or by choosing an individual label.

### Image acquisition and fMRI data analyses

The experimental set-up was similar to a previous study (Park et al., 2014). MRI was performed using a 3 T whole body system (Magnetom VERIO, Siemens, Erlangen, Germany) at the University Hospital of the LMU Munich. The scanner was equipped with a standard TIM head coil (12 elements) and the participant’s head was securely but comfortably fastened by a foam cushions in order to minimize head movements. For acquiring the blood oxygen level dependent (BOLD) functional images, an T2^*^-weighted Echo-Planar Imaging (EPI) sequence was used with the following parameters: repetition time (TR) = 3000 ms, echo time (TE) = 30 ms, flip angle (FA) = 80°, number of slices = 28, slice thickness = 4 mm, inter-slice gap = 0.4 mm, interleaved acquisition, field of view (FOV) = 192 × 192 mm, matrix = 64 × 64, in-plane resolution = 3 × 3 mm. Functional images were obtained in axial orientation, covering the whole cerebrum and dorsal cerebellum. A total of 183 scans were conducted for each participant over all three runs. The functional measurement session lasted approximately 10 min in total. To provide an anatomical reference and to rule out structural abnormalities, a sagittal high-resolution 3D T1-weighted Magnetization Prepared Rapid Gradient Echo (MPRAGE) sequence was performed: TR = 2400 ms, TE = 3.06 ms, FA = 9°, number of slices = 160, FOV = 240 × 256 mm, spatial resolution = 1 mm.

Data were analyzed with SPM8 (Statistical Parametric Mapping^1^). The first five volumes were discarded due to T1 saturation effects. All functional images were realigned (“estimate and reslice”), co-registered (“estimate”; EPI template; Montreal Neurologic Institute, MNI), spatially normalized (“estimate and write”) into standard stereotaxic space using standard SPM8 parameters, re-sliced to 2 × 2 × 2 mm voxels, and smoothed with an [8 8 8] mm full-width at half maximum (FWHM) Gaussian kernel. Each condition was modeled by a boxcar function convolved with the canonical hemodynamic response function. At the first level, *t*-tests were computed for each subject and for each condition vs. the baseline. The baseline of statistical parametric maps in our study is comprised of time periods not defined as conditions in the first-level model (i.e., happy, sad, fearful, and neutral prosody). The individual contrast images for each subject were used for the random-effects second level analysis (Full factorial design with one between-subjects (musicians, non-musicians) and one within-subjects (happy, sad, fearful, neutral prosody) factors). The statistical parametric maps were cluster-level thresholded (cluster-level thresholded at *p*(FDR) < 0.05, starting from *p* uncorrected < 0.01; cluster-size threshold = 300 voxels). Anatomical description was done referring to the AAL atlas (Automated Anatomical Labeling of Activations; Tzourio-Mazoyer et al., 2002).

## Results

### Identification task

A main effect of emotion was revealed by a two-way analysis of variance (ANOVA) with *emotion* as within-subject variable and *group* as between-subject variable, *F*_(3,66)_ = 9.454, *P* < 0.001. Further paired *t*-tests showed that sadness conveyed by speech prosody was as easily identified as neutral voice (0.69 vs. 0.70 in correct identification rate, *P* > 0.05), while happy and fearful voices were equally difficult to be identified (0.48 vs. 0.58 in correct identification rate, *P* > 0.05), as significant differences were only observed between the two categories (i.e., sadness and neutral vs. happy and fear, *P* < 0.05). Importantly, no significant main effect of *group* was observed, *F*_(1,22)_ = 1.546, *p* > 0.05, and no significant two-way interaction was observed either, *F*_(3,66)_ = 1.728, *p* > 0.05. These results seemed to indicate that both musicians and non-musicians are equally capable to identify emotions conveyed in speech prosody, although both groups are better at recognizing sadness as compared to fearful and happy emotions.

### Similarities between groups—conjunction analysis

**Figure 1:**
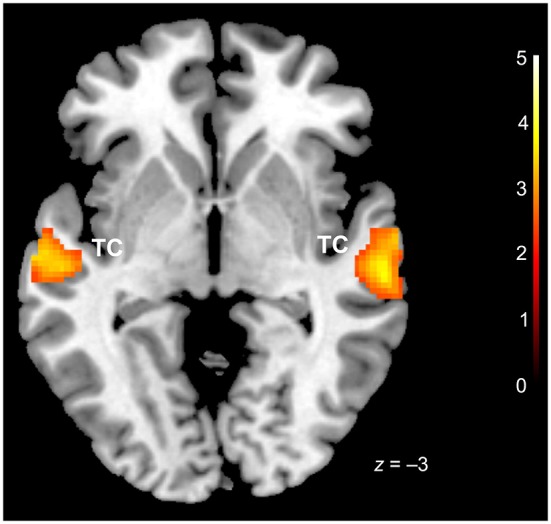
**Conjunction analysis (happiness, sadness, fear vs. baseline)**. TC = temporal cortex. *x* coordinate is in the MNI stereotactic space; cluster-level thresholded at *p* (FDR) < 0.05.

Conjunction analysis (conj. null) for the three basic emotions (happiness, sadness, fear) vs. baseline revealed bilateral activation in the temporal cortex, specifically in middle temporal (BA 21) and STG (BA 22) (Table 1, Figure 1). Possibly due to scanner noises, no distinct increases of activation were found in primary auditory cortices in response to the three emotions.

**Table 1:** **Neurofunctional correlates**.

Brain region	Cluster	kE	Coordinates			***Z***-value
			***x***	***y***	***z***	
*A. Conjunction (all emotions vs. baseline)*
R. superior temporal g., R. middle temporal g. (BA 21, 22)	1	548	66	−10	−6	3.72
L. middle temporal g., L. superior temporal g. (BA 21, 22)	2	359	−64	−16	−2	3.37
*B. Sadness (musicians vs. non-musicians)*
R./L. cingulate g., middle part, R./L. precuneus, R./L. cingulate g., posterior part (BA 23, 31, 7, 29, 30)	1	1591	2	−40	40	3.72
R./L. cingulate g., anterior part, R. middle frontal g., R. superior frontal g.,	2	962	12	44	8	3.64
R. superior frontal g., medial part (BA 9, 10, 46, 32)						

*Note. kE = size in voxels (2 × 2 × 2 mm). R. = right, L. = left, g. = gyrus. The x, y and z coordinates are in the MNI stereotactic space*.

### Differences between groups

**Figure 2:**
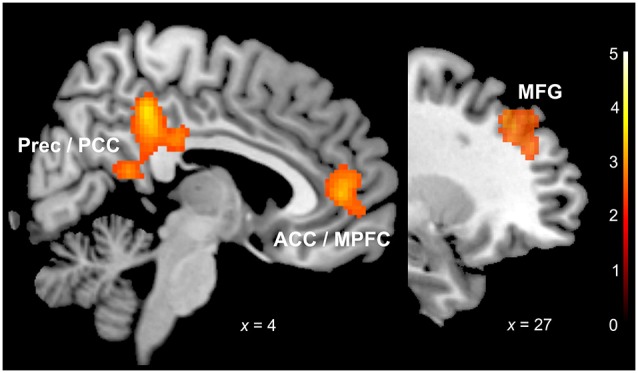
**Sadness (musicians vs. non-musicians)**. ACC: anterior cingulate cortex; MPFC: medial prefrontal cortex; MFG: middle frontal gyrus; Prec: precuneus; PCC: posterior cingulate cortex. *x* coordinate is in the MNI stereotactic space; cluster-level thresholded at *p* (FDR) < 0.05.

We observed significant differences in neural activation between the groups in response to sentences with sad prosody. In response to sad prosody musicians showed a significant increase of activation in the frontal cortex (BA 10, BA 9, 46), ACC (BA 32), posterior cingulate (BA 23, 31) and retrosplenial cortex (BA 29, 30) (Table 1, Figure 2). We did not observe any differences in neural activation between musicians and non-musicians in response to happy or fearful prosody. No increases of activation for non-musicians relative to musicians in response to any of the emotions were found.

## Discussion

The present study revealed similarities and differences between musicians and non-musicians in processing of emotional speech prosody expressing happiness, sadness and fear.

Conjunction analysis for fear, happiness and sadness revealed bilateral activations in temporal cortex, in the middle temporal gyrus (MTG) and the STG in both musicians and non-musicians. These areas are part of an auditory processing stream for categorizing auditory information (Hickok and Poeppel, 2007), including the identification and processing of linguistic and paralinguistic features of speech (e.g., Wildgruber et al., 2005; Schirmer and Kotz, 2006; Ethofer et al., 2012). The STG and the MTG crucially involved in processing emotional prosody (e.g., Mitchell et al., 2003; Leitman et al., 2010; Frühholz and Grandjean, 2013b; Grandjean and Frühholz, 2013) and reliably, the (right) STG is found in studies on emotional prosody processing (Brück et al., 2011; Ethofer et al., 2012; Frühholz et al., 2012; Frühholz and Grandjean, 2013a; Kotz et al., 2013; Belyk and Brown, 2014). It is assumed to play a major role in the early stages of prosody processing and has recently been referred to as the crucial part of the “emotional voice area” (Ethofer et al., 2012).

These common activations suggest that in musicians and non-musicians similar neural mechanisms are recruited for early stage processing of emotional vocal stimuli.

Apart from these similarities we also observed differences in neural responses to emotional speech prosody between the groups. Specifically, musicians showed enhanced activations in several brain areas when responding to sentences spoken with sad prosody, suggesting higher sensitivity in emotion processing. Our observations will be discussed in the context of local neural activations and their assumed associations with subjective representations, being well aware of the conceptual problems when attributing high level cognitive processes to local neural modules or distributed neural networks (Bao and Pöppel, 2012).

We observed activation increases in the musician group in response to sad prosody in right frontal areas, in the middle and SFG (BA 10, BA 9, BA 46). Structural plasticity in right frontal regions has previously been associated with musical training (Hyde et al., 2009). Consistently, models on prosody processing agree in assuming the frontal cortex to play a crucial role in higher levels of prosody processing (see Witteman et al., 2012), specifically in the detection and judgment of emotional speech prosody (see Schirmer and Kotz, 2006). Specifically, the middle frontal gyrus has previously been found to be associated with processing of incongruity of in emotional prosody (Mitchell, 2013) and the detection of sad emotional tone (Buchanan et al., 2000). The stronger activations in right prefrontal areas may thus reflect processes related to the evaluation and categorization of emotional prosody and it might also point to an enhanced sensitivity in the musician group specifically for the sad emotional content of the stimuli.

The increases in frontal activation for the group of musicians in response to sad speech prosody also included the an area comprising the medial part of the SFG and the ACC (BA 10, 32); areas that are both particularly associated with emotional processing, the appraisal and the regulation of emotions (Etkin et al., 2011), and also the induction of emotions (Amodio and Frith, 2006). The ACC is assumed to be part of a network specifically sensitive to monitoring of uncertainty and emotional saliency (Nomura et al., 2003; Cieslik et al., 2013) and the ACC and the medial prefrontal cortex have been specifically associated with the induction of sadness (Beauregard et al., 1998; Mayberg et al., 1999; Bush et al., 2000). Furthermore, the medial prefrontal cortex has been observed to be involved in emotional voice processing (Johnstone et al., 2006; Ethofer et al., 2012), and activation in the ACC has been found to play a special role in processing of emotional prosody (Bach et al., 2008; Belyk and Brown, 2014). We previously found increased activation in prefrontal regions in musicians in response to sadness in a study on musically conveyed emotions (Park et al., 2014) and Escoffier et al. (2013) found activations in the superior frontal cortex and the ACC during the processing of emotions that were expressed in music and through vocalization. The authors assumed that specific social processes might underlie emotion perception in both domains as both the superior frontal cortex and the ACC play a crucial role in mentalizing and other theory of mind (TOM) mechanisms (Escoffier et al., 2013). In fact, the medial prefrontal cortex and the ACC have consistently been associated with empathic processes and perspective taking (Amodio and Frith, 2006; Decety and Jackson, 2006; Etkin et al., 2011) and in particular the medial prefrontal cortex has been termed a “hub of a system mediating inferences about one’s own and other individual’s mental states” (Ochsner et al., 2004). The increased activations in the medial prefrontal cortex and the ACC in the group of musicians in response to sad sentences might thus suggest stronger emotional responses specifically related to the sad prosody of the stimuli. The increases of activation might furthermore point towards specific empathic processes related to the perceived sadness expressed in the stimuli (Harrison et al., 2007).

We also observed stronger activation in musicians in response to sad speech prosody in the posterior cingulate (PCC, BA 23, 31) and the retrosplenial cortex (BA 29, 30). The PCC and retrosplenial region have been associated with internally directed thought and episodic memory functions (Vann et al., 2009; Leech et al., 2012), and they are also involved in the “neural network correlates of consciousness”, playing an important role in cognitive awareness, self-reflection (Vogt and Laureys, 2005) and control of arousal (Leech and Sharp, 2014). The PCC and retrosplenial region are also assumed to be involved in processing of the salience of emotional stimuli (Maddock, 1999) and the emotional content of external information (Cato et al., 2004), specifically of emotional words (Maddock et al., 2003). The increased activation we observed in the PCC and retrosplenial region in response to the sad prosody might, thus, reflect enhanced memory processes as well as increased assessment of emotional saliency of the sad prosodic stimuli and monitoring of arousal.

Some of the areas in which we found activation increases for musicians in response to sad speech prosody can be considered parts of the default mode network (DMN, Raichle et al., 2001; Buckner et al., 2008), specifically the cortical midline structures ACC and PCC and the anterior medial regions of the prefrontal cortex. The DMN shows strong activity at rest and deactivation during tasks that call for external attention. The DMN as a functional system has been associated with processing of self (Zaytseva et al., 2014) and reflects introspective activities and stimulus-independent thought. Such “mentalizing” detaches from the present moment in which stimulus processing takes place (Pöppel and Bao, 2014). Furthermore, the DMN has been associated with induction of emotions, processing of affective saliency (Andrews-Hanna et al., 2010) and with social-emotional processing (Schilbach et al., 2008, 2012), such as attributing mental states to self and others (e.g., Mars et al., 2012).

It may be a puzzling result that the only significant differences between the groups were observed in the neural response to prosody expressing sadness but not in response to the other emotions. However, sadness is consistently found to be one of the emotions that are easiest to recognize (see Thompson et al., 2004). It is characterized by a particularly relevance to social loss (Panksepp, 2005) and may therefore be considered a highly salient and socially relevant signal. Furthermore, the expression of sadness in both music and speech prosody relies on similar acoustic features (Curtis and Bharucha, 2010), which musicians, due to their enhanced acoustic skills, may be able to extract more readily. In a previous study on musical emotions (see Park et al., 2014), we also found that musicians showed stronger neural activations to musical excerpts conveying negative emotions including sadness, and indicated stronger arousal in response to sad music. It was hypothesized that musicians may possibly be at an advantage to respond to the high social saliency of this emotion due to certain gains in social-emotional sensibility. In fact, the social functions and effects of music making have recently received increased attention (Koelsch, 2013) and listening to music has been shown to automatically engage TOM processes such as mental state attributions (Steinbeis and Koelsch, 2009), possibly implying that musicians because of their ongoing training may be particularly experienced in those specific aspects of social-emotional cognition. In fact, there is some empirical indication that musical training does indeed positively influence social emotional and communication development (Gerry et al., 2012) and that musical interventions effectively improve social skills (Gooding, 2011). Thus, a specific increase of social competence and social-emotional sensibility may be one cross-functional benefit of long-term musical training. Assuming these potentially enhanced social cognitive and empathic competences, musicians might thus be more responsive to the high social saliency of sadness in speech prosody. However, while several studies have reported advantages in recognition of emotional speech prosody due to musical training (Thompson et al., 2004; Lima and Castro, 2011), we only observed the difference between musicians and non-musicians in identifying sadness on the neural level, but we did not find any significant differences on the behavioral level. This dissociation between neural responses and verbal reports to sadness supports the general concept to distinguish between the levels of explicit and implicit experience (Pöppel and Bao, 2011). The fact that the difference between the groups was only observed on the neural level suggests that for musicians sadness may be characterized by a unique implicit representation. The neural activations we observed in response to the sad prosody, in particular the activations in the MPFC and other parts of the DMN (Ochsner et al., 2004; Mitchell et al., 2005; Amodio and Frith, 2006), may possibly reflect these social-emotional mechanisms that crucially involve implicit introspective, i.e., self-referential, processes to infer the mental state of the speaker.

Finally, while a transfer effect of musical training to speech processing may mainly depend on acoustic and rhythmic similarities between music and speech (see Jäncke, 2012) temporal mechanisms might constitute another driving force for this cross-functional learning effect. Temporal mechanisms are of utmost importance in coordinating cognitive processes and can be considered to be an anthropological universal (Bao and Pöppel, 2012). Positive learning effects related to temporal training have been observed previously on the level of temporal order thresholds (Bao et al., 2013) of native speaker of the tonal language Chinese who show different thresholds compared to subjects from a non-tonal language environment. Furthermore, temporal mechanisms are crucial for conveying poetry (Turner and Pöppel, 1988) and they can be regarded basic to the expression and experience of music (Pöppel, 1989). Since neuro-imaging studies have shown music and language to rely on similar neural structures (Abrams et al., 2011) and considering the temporal similarities between music and speech it might be suspected that musical training also positively impacts temporal processing, and the observed effects thus may reflect enhanced temporal sensitivity as an effect of inter-modal transfer (Pöppel, 1989, 2009) which might also involve a higher competence to detect sadness in speech.

In conclusion, consistent with a previous study showing differences in emotion processing presumably due to musical training (Park et al., 2014), our study supports the notion that such training also alters the neural processing of distinct emotions conveyed in speech prosody. In particular, while musicians and non-musicians do not differ in their performance in recognizing sadness in speech, they process this particular emotion significantly differently on the neural level. Musicians show distinct increases of neural activations only in response to the sad prosody, possibly due to a higher affective saliency that the sentences spoken with sad intonation might possess. Our results imply that the cross-modal transfer effects of musical training go beyond auditory processing and explicit emotional recognition skills; we suggest that such training may also impact the empathic aspects in human communication.

## Conflict of interest statement

The authors declare that the research was conducted in the absence of any commercial or financial relationships that could be construed as a potential conflict of interest.

## Footnotes

^1^http://www.fil.ion.ucl.ac.uk/spm
